# Comparative dissection of transcriptional landscapes of human iPSC-NK differentiation and NK cell development

**DOI:** 10.1093/lifemedi/lnae032

**Published:** 2024-09-06

**Authors:** Li Zhang, Taylor M Weiskittel, Yuqing Zhu, Dixuan Xue, Hailing Zhang, Yuxuan Shen, Hua Yu, Jingyu Li, Linxiao Hou, Hongshan Guo, Zhijun Dai, Hu Li, Jin Zhang

**Affiliations:** The Bone Marrow Transplantation Center of The First Affiliated Hospital &Liangzhu Laboratory, Zhejiang University School of Medicine, Hangzhou 310012, China; Center for Stem Cell and Regenerative Medicine, Department of Basic Medical Sciences, Zhejiang University School of Medicine, Hangzhou 310058, China; Center for Individualized Medicine, Department of Molecular Pharmacology & Experimental Therapeutics, Mayo Clinic, Rochester, MN 55905, USA; Center for Stem Cell and Regenerative Medicine, Department of Basic Medical Sciences, Zhejiang University School of Medicine, Hangzhou 310058, China; Center for Stem Cell and Translational Medicine, School of Life Sciences, Anhui University, Hefei 230601, China; Center for Stem Cell and Regenerative Medicine, Department of Basic Medical Sciences, Zhejiang University School of Medicine, Hangzhou 310058, China; Department of Breast Surgery, The First Affiliated Hospital, Zhejiang University School of Medicine, Hangzhou 310012, China; The Bone Marrow Transplantation Center of The First Affiliated Hospital &Liangzhu Laboratory, Zhejiang University School of Medicine, Hangzhou 310012, China; Center for Stem Cell and Regenerative Medicine, Department of Basic Medical Sciences, Zhejiang University School of Medicine, Hangzhou 310058, China; The Bone Marrow Transplantation Center of The First Affiliated Hospital &Liangzhu Laboratory, Zhejiang University School of Medicine, Hangzhou 310012, China; Center for Stem Cell and Regenerative Medicine, Department of Basic Medical Sciences, Zhejiang University School of Medicine, Hangzhou 310058, China; Center for Stem Cell and Regenerative Medicine, Department of Basic Medical Sciences, Zhejiang University School of Medicine, Hangzhou 310058, China; Center for Stem Cell and Regenerative Medicine, Department of Basic Medical Sciences, Zhejiang University School of Medicine, Hangzhou 310058, China; Center for Stem Cell and Regenerative Medicine, Department of Basic Medical Sciences, Zhejiang University School of Medicine, Hangzhou 310058, China; The Bone Marrow Transplantation Center of The First Affiliated Hospital &Liangzhu Laboratory, Zhejiang University School of Medicine, Hangzhou 310012, China; Institute of Hematology, Zhejiang University, Hangzhou 310058, China; Department of Breast Surgery, The First Affiliated Hospital, Zhejiang University School of Medicine, Hangzhou 310012, China; Center for Individualized Medicine, Department of Molecular Pharmacology & Experimental Therapeutics, Mayo Clinic, Rochester, MN 55905, USA; The Bone Marrow Transplantation Center of The First Affiliated Hospital &Liangzhu Laboratory, Zhejiang University School of Medicine, Hangzhou 310012, China; Center for Stem Cell and Regenerative Medicine, Department of Basic Medical Sciences, Zhejiang University School of Medicine, Hangzhou 310058, China; Center of Gene and Cell Therapy and Genome Medicine of Zhejiang Province, Hangzhou 310000, China; Institute of Hematology, Zhejiang University, Hangzhou 310058, China

**Keywords:** iNK, single-cell RNA-seq, STAT5A

## Abstract

Clinical and preclinical research has demonstrated that iPSC-derived NK (iNK) cells have a high therapeutic potential, yet poor understanding of the detailed process of their differentiation *in vitro* and their counterpart cell development *in vivo* has hindered therapeutic iNK cell production and engineering. Here we dissect the crucial differentiation of both fetal liver NK cells and iNK cells to enable the rational design of advanced iNK production protocols. We use a comparative analysis of single-cell RNA-seq (scRNA-seq) to pinpoint key factors lacking in the induced setting which we hypothesized would hinder iNK differentiation and/ or functionality. By analyzing key transcription factor regulatory networks, we discovered the importance of *TBX21*, *EOMES*, and *STAT5A* in the differentiation timeline. This analysis provides a blueprint for further engineering new iPSC lines to obtain iNK cells with enhanced functions. We validated this approach by creating a new line of STAT5A-iPSCs which can be differentiated to STAT5A-expressing macrophages with both NK cell and macrophage features such as perforin production, phagocytosis, and anti-tumor functions.

## Introduction

Induced pluripotent stem cells (iPSC)-derived innate immune cells, such as natural killer (NK) cells and macrophages, have shown great promise in clinical and preclinical settings [1, 2]. Various strategies have been attempted to regenerate NK cells from human pluripotent stem cells (hPSCs). A pioneering study generating CD56^+^ NK cells from hESCs relied on OP9 cell coculture and the addition of fetal bovine serum (FBS) and cytokine cocktail [3]; To eliminate the adverse impacts of xenogeneic feeder cells, a complete feeder cell‐ and serum‐free culture medium was introduced with the “spin embryoid body (EB)” method [4, 5]. Three-dimensional structure with stromal cells is helpful for hemogenesis and lymphogenesis, and consequently, an EB-free and organoid aggregate method for NK cell production from hPSCs was developed with stable and high efficiency [6]. However, there are still many obstacles that must be overcome before iPSC innate immune therapies can be widely translated to the bedside. Among them, the absence of biological understanding of the differentiation and developmental processes has prevented efficient and reproducible NK cell production via iPSC differentiation, and further exploration is needed on several fronts. Firstly, the determinants of what makes a productive iPSC clone have yet to be revealed. Secondly, much of the regulatory dynamics that occur during *in vitro* immune cell differentiation procedures have yet to be captured or understood. Modern single-cell sequencing technologies present unique opportunities to understand this “black box”, and information gleaned from comparative analysis between *in vivo* and *in vitro* datasets can act as a rational design framework for enhanced directed differentiation.

Natural immune differentiation branches to produce several immune cell populations, but in directed iPSC differentiation a single-cell type is often desired. The first point of rational design intervention is thus to compare desired populations to undesired “byproduct” cell types and capitalize on the identified differences to produce a higher percentage of desired populations while reducing byproduct contamination. A second opportunity for rational design is to take inspiration from the natural progressions found *in vivo*. Previous studies have compared the iPSC differentiation of NK cells to adult blood and umbilical cord blood NK cells or macrophages [7]. However, new evidence indicates that iPSC differentiation of immune cells is more similar to fetal immune cell differentiation and is not readily comparable to the processes present in adult bone marrow [8]. Due to sample availability, a head-to-head comparison of iPSC to fetal differentiation has not been performed, but recently ground-breaking single-cell RNA-sequencing (scRNA-seq) from the first trimester of human fetal development has been conducted [9]. For this reason, we were able to directly juxtapose fetal *in vivo* and iPSC *in vitro* NK differentiation for the first time.

To visualize and understand the complexities of these two settings, we took a systems biology approach. Gene regulatory network (GRN) analysis has enabled cell fate reprogramming and conversion in several other biological contexts [10]. Transcription factors (TFs) have been established as the primary driving force of cellular differentiation and state which necessitates a broad evaluation of TF activation in these immune cell contexts. We thus focused on creating TF GRN for iPSC-differentiated innate immune cells *in vitro* and for the fetal innate immune cell in development *in vivo* for efficient interpretable comparison. Once the key discerning TFs within the GRNs are identified, genetic or pharmacologic interventions can then push *in vitro* differentiation to more faithfully recapitulate *in vivo* biology. In this way, we establish a TF-driven “Build–Test–Learn” cycle applicable to cellular engineering and synthetic biology endeavors far beyond immunotherapies.

## Results

### Intrinsic iPSC priming toward hematopoietic lineages dictates differentiation efficiency

Engineered iPSC-derived NK cells (iNK) have displayed therapeutic potential with high anticancer cytotoxicity [3, 11, 12]. For this reason, we focused on iNK cells as our population of interest. We started by deriving iPSCs from healthy donor peripheral blood mononuclear cells (PBMC) using non-integrating episomal vectors encoding reprogramming factors OCT3/4, SOX2, KLF4, L-MYC, and LIN28.

**Figure 1.**
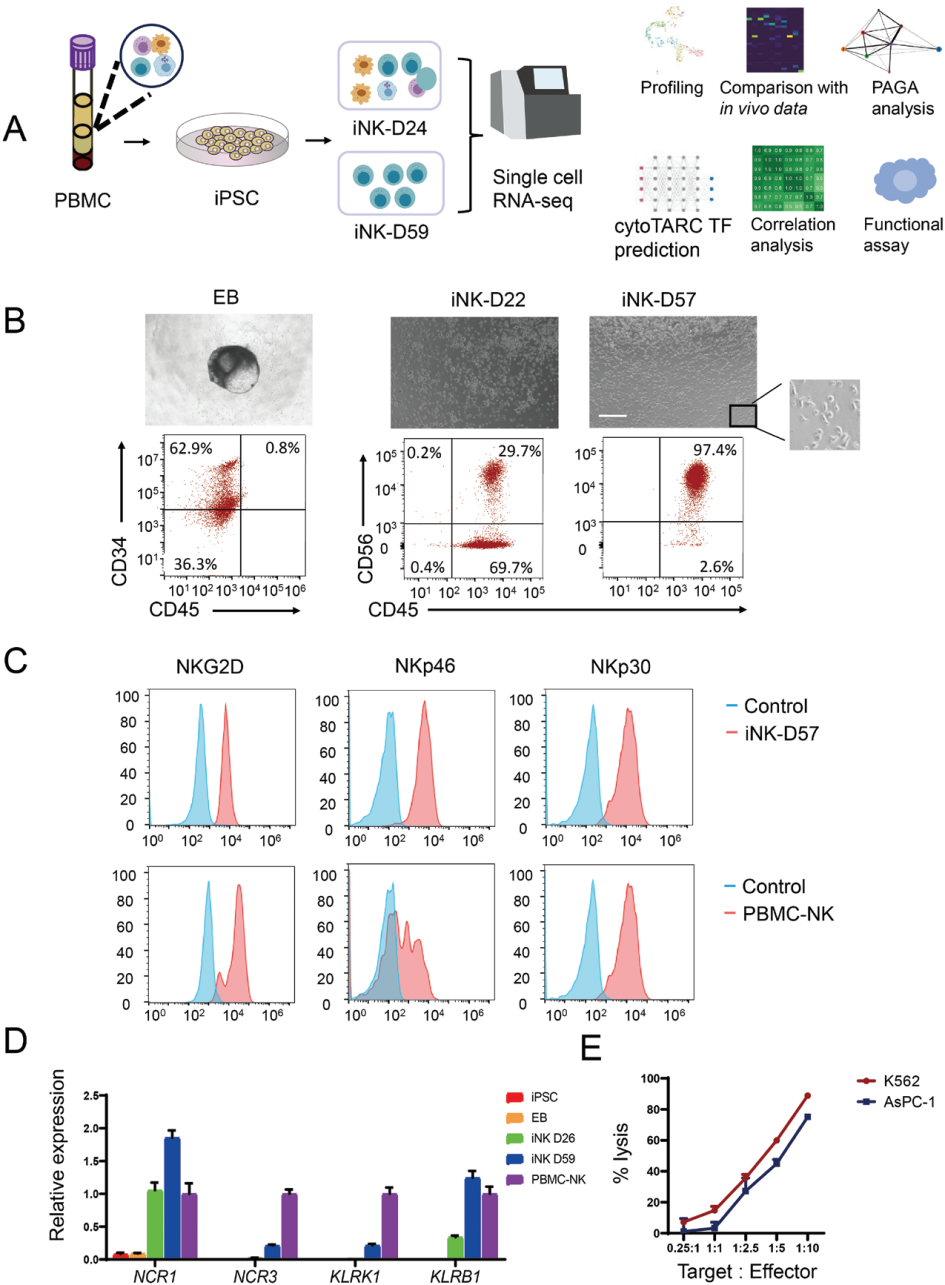
**Differentiation of iPSCs into NK cells.** (A) Schematics show the procedures of differentiation from iPSCs to NK cells and subsequent bioinformatics analysis. (B) Representative microscopic pictures show cell morphology at different differentiation stages and corresponding flow cytometry analysis. (C) Flow cytometry analysis of typical NK receptors with iPSC-derived NK at Day 57 (iNK-D57) and PBMC-NK. (D) qRT–PCR shows key NK cell marker gene expression at different stages of differentiation. (E) iNK *in vitro* killing assay against luciferase-expression K562 and AsPC-1 cells with various target ratios.

The iPSC to iNK differentiation protocol was completed on 5 iPSC clones, and flow cytometry revealed that a varying efficiency of iPSC differentiation to hematopoietic lineage (CD34^+^) (Fig. S1A). To understand the driving forces of this phenomena, we compared the transcriptomics of the best-performing clone iPSC 3-8 against the other clones such as iPSC1-1. We found that iPSC 3-8 had increased expression of TFs known to specifically regulate NK differentiation, such as EOMES when compared with iPSC 1-1 (Fig. S1B). Moreover, Kyoto Encyclopedia of Genes and Genomes (KEGG) analysis showed that iPSC 3-8 was enriched in MAPK signaling-related transcripts (Fig. S1C) which was consistent with MAPK’s known role in hematopoiesis [13]. iPSC 3-8 was additionally enriched in other stemness and hematopoiesis pathways indicating that at baseline the 3-8 clone had existing polarity primed towards hematopoietic lineages. We thus focused on the most efficient clone, 3-8, for the following analysis (Fig. 1A).

Subsequently, we established a modified protocol of lymphoid/NK differentiation as previously described [13]. To characterize the temporal differentiation of iNK cells, we collected iNK differentiation cultures at EB, Days 22 (iNK-D22), and 57 (iNK-D57) for flow cytometry analysis (Fig. 1B). At Day 22, not only 99.4% of cells were found to be CD45^+^ lymphoid cells but also 29.7% were CD45^+^ CD56^+^ NK cells. The percentage of CD56^+^ NK increased to 97.4% at Day 57 (Fig. 1B). On Day 57 iNK cells showed positivity for NKG2D, a major cytotoxicity activation receptor and the natural cytotoxicity receptors NKp46 and NKp30. When compared to PBMC-NK cells, iNK-D57 cells showed almost equivalent increases over control cells on these key markers. However, iNK-D57 populations differed from PBMC-NK cells in that expression of these key markers, which showed a unimodal distribution, while PBMC-NK distributions followed more complex patterns, indicating that the PBMC-NK cells were a more heterogenous group (Fig. 1C). Meanwhile, other activated receptors such as *KLRC2* and *KLRC3* were detected in the RNA-seq data (Fig. S1K).

To validate our modified protocol, we probed for the expression of known lineage markers at key differentiation milestones. The pluripotency genes (*NANOG*, *SOX2*, and *POU5F1*) specifically expressed at the iPSC stage [14], and in the EB the hematopoietic signatures of *CD34* and *GATA6* were increased as compared to the earlier iPSC stage (Fig. S1D and S1E). The expression of hematopoietic stem cell (HSC) regulator *GATA2* increased at Day 26 (Fig. S1E), and this gene has been reported to be a key regulator of human NK cell maturation [15]. As differentiation progressed, mature NK cell-specific signatures began to be expressed at Days 26 and 59 including natural killer cytotoxicity and activating receptors (Figs. S1F and 1D). The iNK population also began to show high expression of NK immunoglobin-like receptors (KIRs) at Day 59 (Fig. S1G) and interleukin receptors such as *IL21R* at Day 26 and *IL2RG* at day (Fig. S1H) which have been shown to stimulate NK *ex vivo* expansion [16, 17]. Surprisingly, Day 59 NK cells expressed higher *KIR*s (*KIR2DL2* and *KIR2DL4*) and interleukin receptor levels (*IL2RG* and *IL21R*) than adult PBMC-NK cells. Other inhibitory or activating receptors such as *KLRC1* and *KLRC2* were also highly expressed in iNK at Day 59 (Fig. S1I). We also detected that the expression of *CD38* dropped markedly in iNK at Day 59, which may be beneficial to iNK activity as CD38 knockdown can contribute to enhanced NK cytotoxicity (Fig. S1J) [18]. On the other hand, the expression of *CISH*, a negative NK regulator, was higher in iNK-59 as compared to PBMC-NK which could dampen activity in iNK-59 cells (Fig. S1J) [11]. To ensure that the expression of these markers conferred functional activity, we confirmed the iNK killed AsPC-1 and K562 cells *in vitro* by performing lytic capability assays (Fig. 1E).

### iNK cells closely resemble NK cells after undergoing dynamic transcriptional changes through differentiation

**Figure 2.**
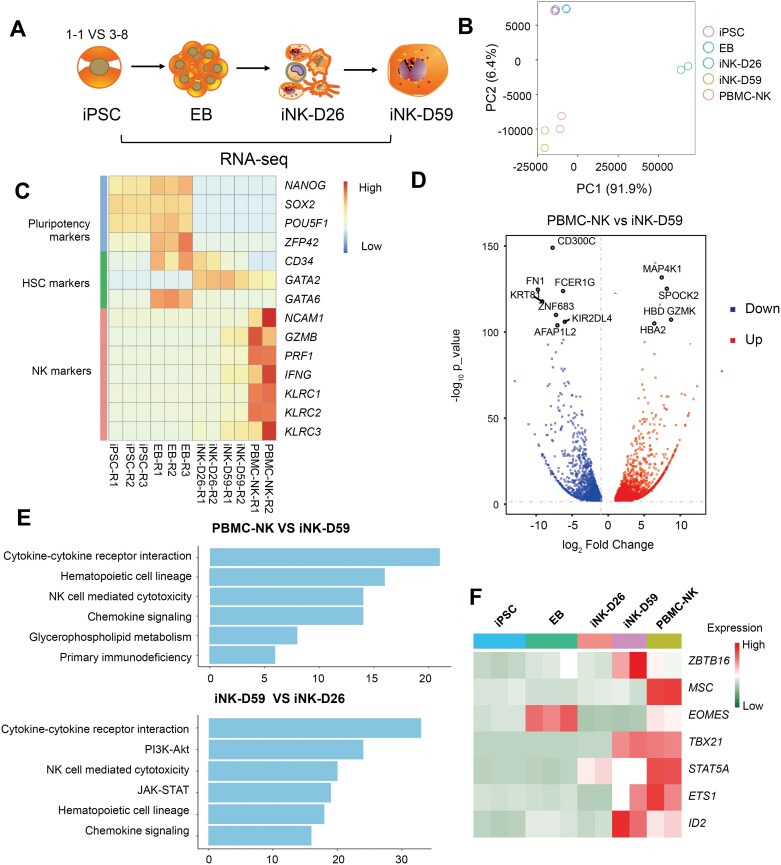
**Transcriptional profiling of iNK cells.** (A) The schematic of RNA-seq sample collection. (B) Principal component analysis (PCA) of iPSCs, EB, differentiated NK cells, and primary CD56 positive NK cells. (C) Heatmap of gene expression of selected representative NK-related genes. (D) The volcano plot shows differentially expressed genes between PBMC-NK and iNK-D59. (E) Top GO terms enriched in genes up-regulated in some pairwise comparison samples. (F) The heatmap shows the gene expression of selected TF genes.

To gain a deeper understanding of the iNK differentiation process, we performed bulk RNA-seq analysis at each key differentiation stage (Fig. 2A). As expected, all six samples from the iPSC and EB stages were closely associated, and the late-stage iNK cells were closest associated with PBMC-NK cells (Fig. 2B). The overall gene expression patterns of samples from the same differentiation stage were similar, and comparisons between iPSC, EB, iNK at Day 26 and Day 59 showed temporal changes in gene expression (Fig. 2B and 2C). For example, pluripotency genes were shut down at the EB stage, hematopoietic markers increased in the EB stage, and many NK receptors and activation markers gradually turned on between Day 26 and Day 59. These results thus confirmed that a successful differentiation protocol had been established.

In comparing iPSC 3-8 to iNK-D59, decreased pluripotency markers were seen (Fig. S2A), and this trend of decreasing pluripotency and increasing lineage specificity was appreciated at the intermediate stages as well with iNK-D26 enrichment of lineage-specific genes when compared to EB stage (Fig. S2B). Finally, we compared the two iNK time points and saw that iNK-D26 was likely still in a precursor state because iNK-D59 exhibited increased expression of *GZMB*, *PRF1*, and *IL2RB* (Fig. S2C).

To ascertain if the iNK cells possessed a similar cell state to primary NK cells, we further compared iNK at Day 59 with NK purified from PBMC. iNK exhibited reduced expression of *GZMK* and decreased enrichment in the NK cell-mediated cytotoxicity pathway, iNK-D59 showed strong enrichment of NK cell-related functions such as “cytokine–cytokine receptor interaction” and “NK cell-mediated cytotoxicity” compared to iNK-D26, and PBMC-NK had more significant enrichment of these pathways. (Fig. 2D and 2E). Several other NK-related markers showed high similarity in iNK-D59 and PBMC-NK samples (Fig. S2D). Furthermore, TFs connected with NK activation and development such as *ZBTB16*, *TBX21*, and ID*2* were highly expressed in the differentiated NK population [19]. Surprisingly, *EOMES* was most highly expressed at the EB stage (Fig. 2F), suggesting its unique role in the dynamic differentiation process. These results demonstrate that even though there are slight differences reflecting the higher maturity of PBMC-NK cells, the iNK cells can largely recapitulate the transcription program of their primary cell counterparts.

### Single-cell profiling reveals multilymphoid progenitors give rise to iNK and induced mast cells during *in vitro* differentiation

**Figure 3.**
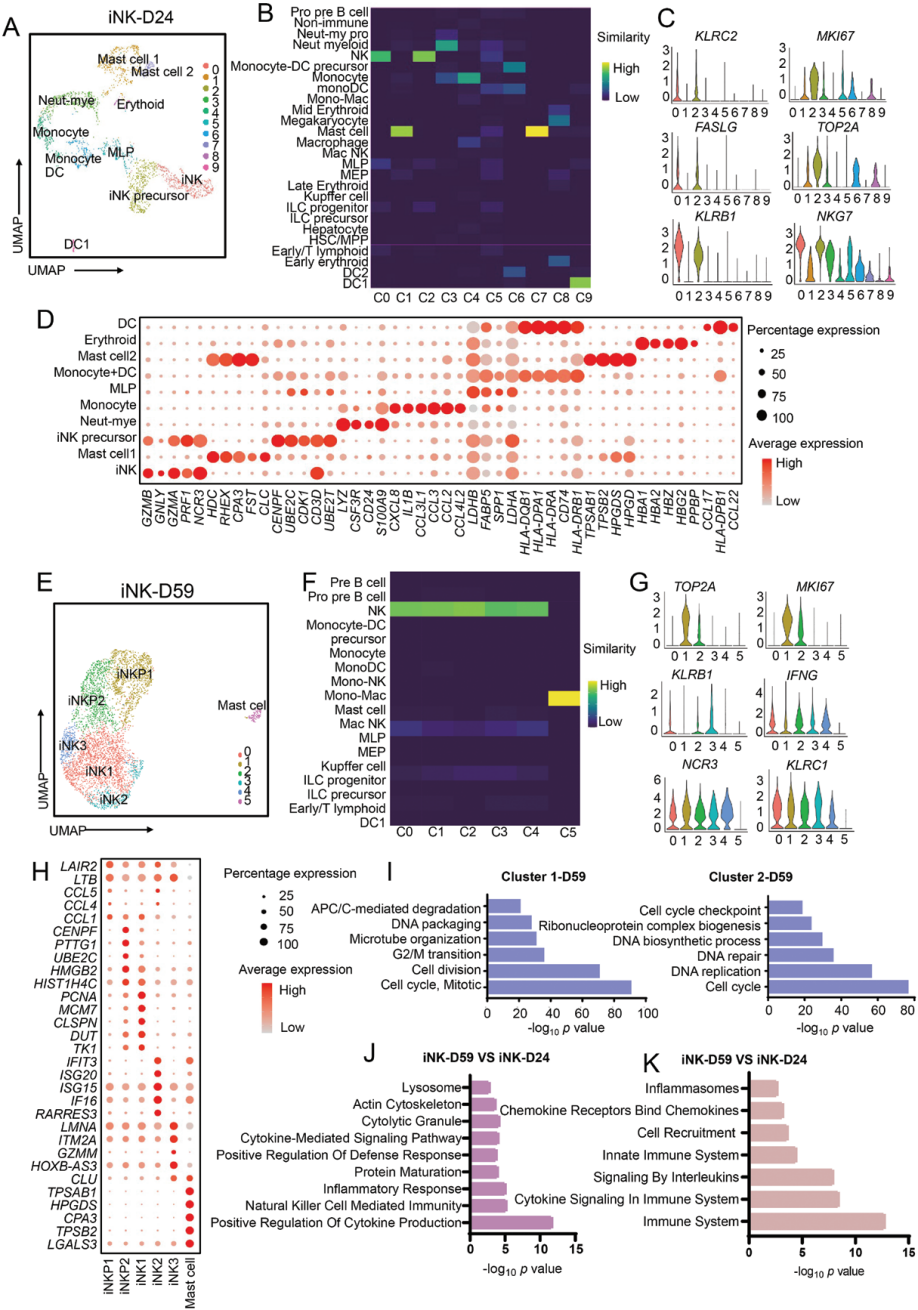
**Single-cell transcriptome profiling of iPSC-derived NK at Days 24 and 59.** (A) UMAP visualization of ten iPSC-derived NK-related cell clusters at differentiation Day 24. (B) Heatmap shows the transcriptome similarity between *in vitro* differentiation Day 24 and *in vivo* development cell clusters. Colors represent the similarity score. (C) Violin plots show the expression of featured genes expressed in different cell clusters at differentiation Day 24. (D) Dot plots show the expression level of the top five differentially expressed genes of each cell cluster at differentiation Day 24. Colors represent the average expression and size encodes the proportion of gene-expressing cells. (E) UMAP visualization of ten iPSC-derived NK-related cell clusters at Day 59. (F) Heatmap shows the transcriptome similarity between *in vitro* differentiation Day 59 and *in vivo* development cell clusters. Colors represent the similarity score. (G) Violin plots show the expression of featured genes expressed in different cell clusters at differentiation Day 59. (H) Dot plots show the expression level of the top five differentially expressed genes of each cell cluster at differentiation Day 59. Colors represent the average expression and size encodes the proportion of gene-expressing cells. (I) The major GO Biological Process terms in which cell cycle-specific genes are enriched for c1 and c2 clusters in iNK-D59. (J) Top GO terms enriched in genes up-regulated in iNK-D59 compared with iNK-D24. (K) Top reactome pathways enriched in genes upregulated in iNK-D59 compared with iNK-D24.

After successfully characterizing the broad changes that occur in iNK differentiation, we then examined the populations present at Days 24 and 59 (similar time points with flow cytometry and RNA-seq analysis) using scRNA-seq. After quality control and doublet exclusion, a total of 2453 cells at iNK-D24 and 4939 cells at iNK-D59 were captured. At Day 24, 10 distinct clusters were found, and we identified these clusters using the SciBet algorithm and reference data from *in vivo* fetal liver development scRNA-seq data between 7 and 17 postconceptional weeks (PCW) [9] (Fig. 3A and 3B). To confirm SciBet predictions, we examined the expression of known marker genes and the top differentially expressed genes in each cluster (Fig. 3C and 3D). In all strongly predicted clusters (C0, C1, C2, C3, C7, and C9), marker genes were consistent with the SciBet assignment (Fig. 3B–D).

iNK-related clusters were distinguished by the enrichment of *NCAM1* (CD56), *GZMB, PRF1, GATA3*, and lymphoid-specific gene *CD7*, while myeloid clusters highly express *CD14* and *LYZ* (Figs. 3A, 3B and S3A). Interestingly, we found that cluster 5 highly expresses NK marker genes, together with myeloid-specific genes. Due to this duality, we assigned this cluster as a multilymphoid progenitor (MLP) which serves as a lymphoid priming progenitor in the HSC compartment [20]. When comparing the iNK clusters, we noted that Cluster 2 had increased expression of *KLRB1* and was closely related to both Cluster 0 and Cluster 5 (Fig. 3A and 3B). This indicates that Cluster 2 is likely an iNK precursor i.e. a stepping stone between MLP (C5) and a more mature iNK phenotype (C0). Additionally, Cluster 2 highly expressed the DNA replication-related gene DNA topoisomerase II Alpha (*TOP2A*) and the proliferation markers *NKG7*, marker of proliferation Ki-67 (*MKI67*) further confirming its role as a proliferative precursor to the iNK population (Fig. 3B and 3D). Cluster 1 and Cluster 7 did not express CD56 or CD14 but were enriched in mast cell markers such as *HDC* and *CPA3* (Fig. 3D). The presence of two distinct maturing immune cell types indicated that multiple lineage specification was underway during the early phase of differentiation.

To further characterize this picture of iNK differentiation, we utilized partition-based graph abstraction (PAGA) analysis to complete trajectory analysis. PAGA analysis on the day 24 sample showed a separation of lymphoid and myeloid lineages (Fig. S3D). The MLP cluster was located centrally in the PAGA trajectory map and showed connections with iNK and myeloid cell types. This was consistent with other works showing that MLP not only gives rise to all lymphoid cell types, but also myeloid cell types like monocytes, macrophages and DCs [21].

### NK cells with a gradient of maturity remained after differentiation and are the source of mature iNK cells

The iNK-D59 sample was analyzed in the same manner as the iNK-D24 sample in concordance with the flow cytometry results, the iNK-D59 sample contained 97.6% iNK cells spread across with five subclusters that all highly express *NCAM1* (*CD56*), *PRF1* and *GZMB*, and the rest population was *KIT* positive mast cells (Figs. 3E, 3F and S3B). To explore the NK cell subpopulations, we generated differentially expressed genes for each cluster and used these gene lists for pathway analysis. The *TOP2A*, *MKI67*, cell cycle-related pathways, and other proliferative markers were enriched in the C1 and C2 clusters compared to the remaining clusters. The mature NK marker *KLRB1* was also downregulated in these clusters, therefore we designated these clusters as less mature progenitor populations (iNKP1 and iNKP2) (Fig. 3G). Importantly, these clusters are further differentiated into NK cells than the MLP (C5) population in the iNK-D24 sample as identified by the SciBet similarity measurements (Fig. 3B and 3F).

The remaining three iNK clusters showed enrichment of immune-related pathways like NK cell activation and immune signaling pathways (Fig. S3C). The three more mature clusters of NK cells expressed more specific pathways related to chemokine, interferon, and immune cell activation. This indicates that within these more mature clusters, there were heterogeneous cell states present (Fig. 3H and S3C). PAGA analysis at iNK-D59 showed that iNKP1 and iNKP2 were closely connected at the beginning of the trajectory with the next earliest population being iNK2 (Fig. S3E).

**Figure 4.**
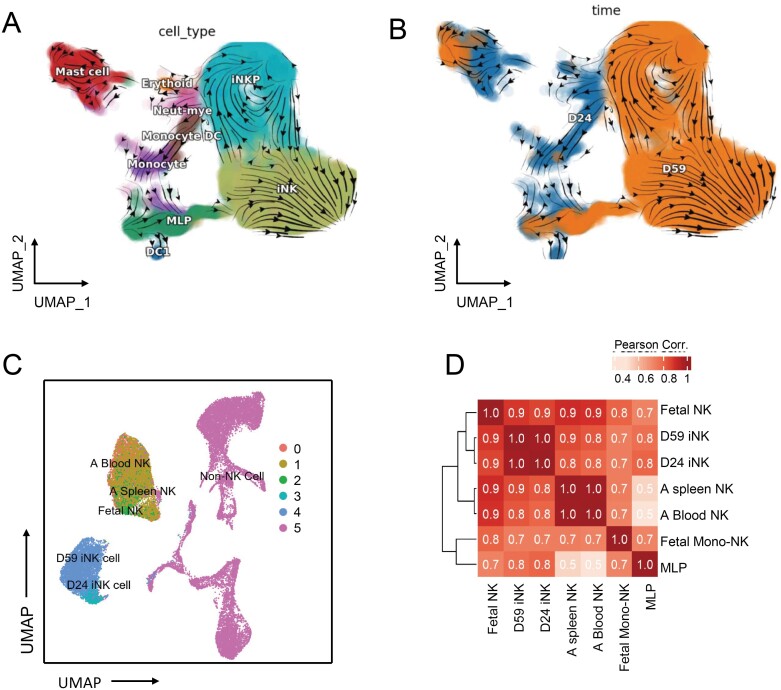
**The adult, fetal, and differentiated NK cells.** (A) RNA velocity visualization of iNK development, the arrows indicated the population flow direction. (B) UMAP visualization of iNK at Days 24 and 59 with pseudotime analysis. (C) UMAP visualization of *in vivo* developed fetal, adult and *in vitro* differentiated NK cells with merged scRNA-seq data. (D) Heatmap of Pearson correlation matrix among *in vitro* iNK and *in vivo* NK cells. Colors indicate the correlation coefficient.

Looking across the two time points, we found that the iNK population in the iNK-D24 sample expressed low levels of *KLRD1* and high levels of *KIT*, and the iNK-D59 sample showed the opposite trend (Fig. S3F and S3G). Next, Gene Ontology (GO) terms and reactome pathway analysis further confirmed that iNK-D59 had more obvious enrichment of NK features and functional pathways than iNK-D24, such as “natural killer cell mediated immunity” and “immune system” (Fig. 3J and 3K). These markers suggest that the cells of iNK-D59 were more mature with increased cytolytic activity [22]. To dissect the sequential relationship among each subpopulation, we merged scRNA-seq data of iNK Days 24 and 59 for further analysis. RNA velocity showed a strong directional flow from iNKP to iNK cells, which originated in a small group of MLP. Besides, the myeloid lineages showed separated flow with iNK cells (Fig. 4A and 4B). Together, these analyses demonstrate that there is a sequential differentiation trajectory from progenitors to less mature iNKs, and finally to mature iNKs.

### iNK cells correlated with *in vivo* fetal and adult NK cells but remained a distinct separate phenotype

The scRNA-seq from both iNK time points was merged with the fetal liver and adult NK scRNA-seq samples [23] so that the relationship between iNK and their counterparts *in vivo* could be understood. On UMAP representation, iNK cells from both time points merged into one discrete cluster while the *in vivo* NK cells from both adult and fetal tissue clustered separately (Fig. 4C). Both remained distinct from other populations of immune cells (Fig. 4C). These differences were quantified using a Pearson correlation between each cell type. Overall, the NK cells from each dataset clustered together thus recapitulating the separation visualized on UMAP (Fig. 4D). More specifically, the two iNK time points showed a strong correlation with each other as did the adult spleen and blood NK cells (Fig. 4D). Besides, iNK at both Days 24 and 59 showed a similar level of agreement with the fetal NK than adult (spleen and blood) NK cells (Fig. 4D). When looking at the differentially expressed genes, we found iNK-D59 had higher gene expression in translation and macromolecule biosynthesis, compared to iNK-D24, suggesting the more matured iNK had higher metabolic activity (Fig. S4A and S4B). Moreover, iNK-D24 and fetal NK were more enriched in cell cycle-related genes, suggesting more active proliferation of these cells, while the adult NK cells were more enriched in defense response genes, indicating their more matured immune functions (Fig. S4A and S4B).

### Characterization of key transcription factors and gene regulatory network of *in vivo* fetal NK differentiation identifies targets for enhanced iPSC differentiation toward iNKs

**Figure 5.**
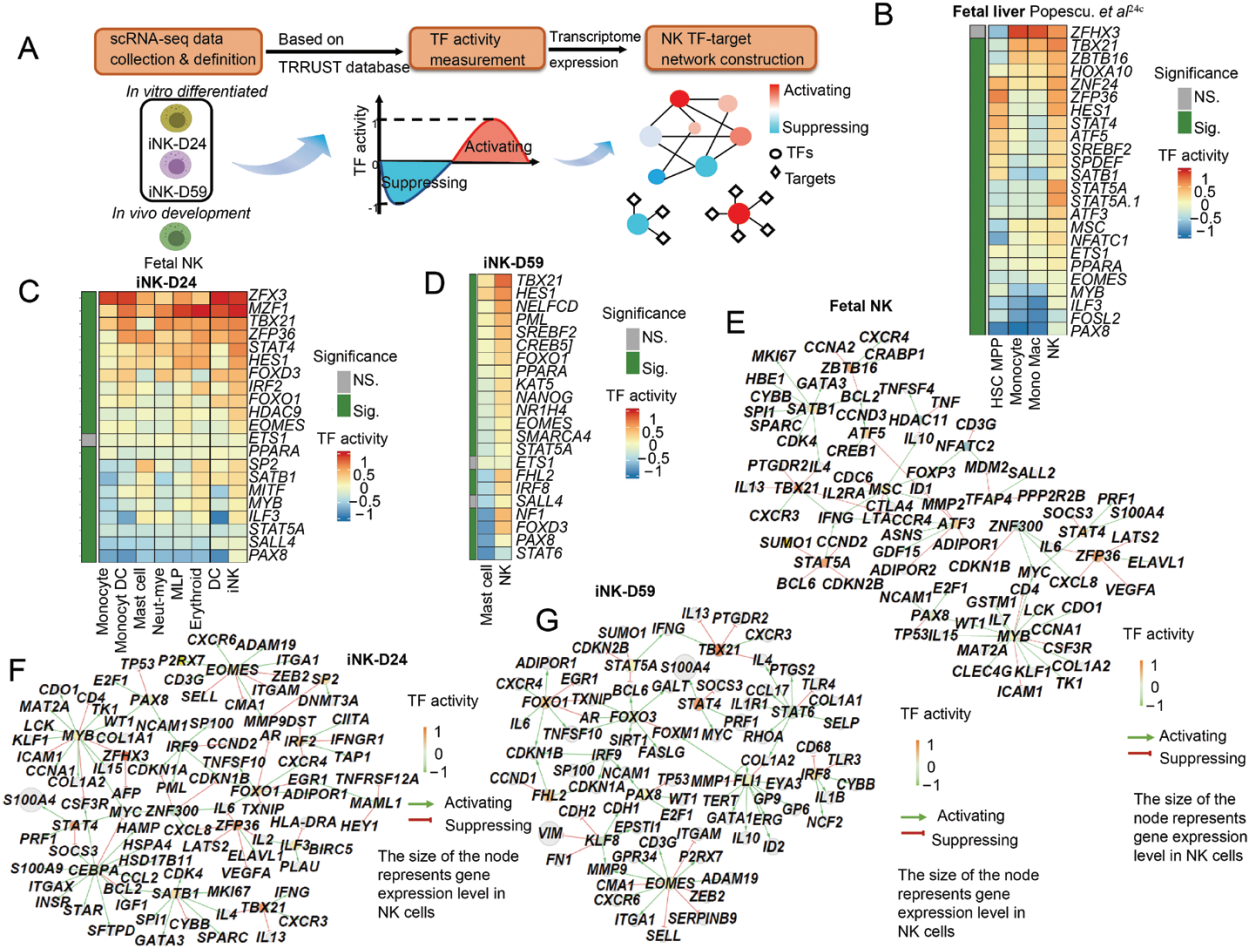
**Validation and assessment of TF-coding genes.** (A) Schematic diagram of cytoTRAC analysis and TF network construction. (B) Heatmap shows the positive regulation TF activity in NK cells compared with HSC and myeloid lineages based on scRNA-seq data from Popescu et al. [23]. Colors represent the average TF activity. (C) Heatmap shows the positive regulation TF activity at Day 24 differentiated NK cells and other-related clusters. Colors represent the average TF activity. (D) Heatmap shows the positive regulation TF activity at Day 59 differentiated NK cells compared with mast cells. Colors represent the average TF activity. (E–G) The correlation network of TFs of NK cells. All TFs in the NK cluster were used to construct network. Colors indicate TF regulation activity.

As NK differentiation from iPSCs mimics *in vivo* fetal NK development, the developmental landscape depicted and the key transcription factor or epigenetic factors identified to instruct NK cell fate decision can provide important guidance in further engineering the NK cells. To quantify the transcription factors activity, we adapted the process of template matching for differential expression by defining templates using the TRRUST database [24] and examining how well each cell’s transcriptome matched the expected pattern for a given TF (Fig. 5A). To determine the TF activities in the context of *in vivo* differentiation, we used human fetal liver scRNA-seq data [9]. In the hematopoietic stem cells (HSC) and multipotent progenitors’ population, *SPI1* and *GATA3* exhibited increased activity which was consistent with the previous studies revealing that they play critical roles in mediating hematopoietic lineage specification and maintenance [25, 26] (Fig. S5A). In contrast, *ZBTB16, TBX21*, and *STAT5A* exhibited more specific activities in the NK population (Figs. 5B, S5A and S5B). Importantly, *TBX21* and *EOMES* had increased activities in both iNK-D24 and iNK-D59. Both of these TFs play central roles in NK cell development and maturation (Fig. 5B–D) [27]. Conversely, some TFs were negatively associated with iNK cells compared with the other counterpart cells (Fig. S5C and S5D).

To better appreciate the macroscopic regulatory network driving NK cell fate, we performed a network analysis of the key up and downregulated TFs. In fetal liver NK cells, *TBX21*, *MSC*, *STAT5A*, and *ZBTB16* were key hub TFs that drove target gene expression such as *CXCR3*, *IFNG*, *IL2RA*, and *CXCR4* (Fig. 5E). In the iNK populations of Day 24 samples, we observed *TBX21* regulating the same target genes, as well as additional hub TFs such as *EOMES* in regulating *CD3G*, *CXCR6* and *ITGA1*, and *IRF9* in regulating *NCAM1* (CD56), whereas some NK hub TFs and their networks were missing such as *ZBTB16* and *MSC* (Fig. 5F). Comparing the two timepoints, iNK-D24 and iNK-D59 shared the networks of *TBX21* and *EOMES* (Fig. 5F and 5G). On the other hand, the iNK-D24 retained some network of progenitor cells such as *MYB* and *FOXO1*, whereas iNK-D59 gained some distinct features such as *FHL2* and *FOXO3*, suggesting a dynamic progressive transcriptional landscape during iNK differentiation. Together, these results demonstrated a shared gene regulatory network and hub TFs, and the distinct ones who are potential targets for further iNK cell engineering.

### STAT5A enhanced the phagocytosis and cytotoxicity functions of iPSC-differentiated macrophages

**Figure 6.**
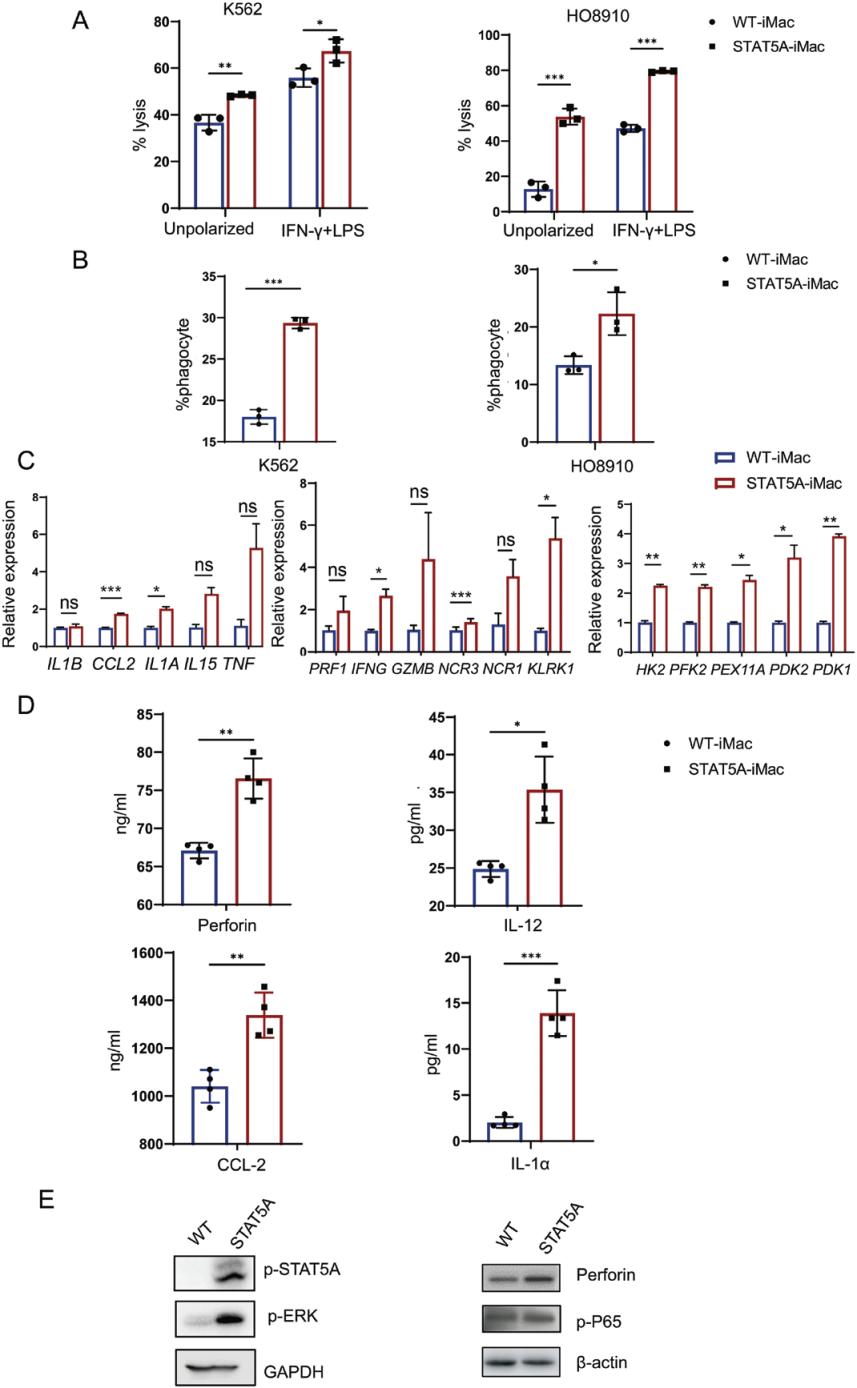
**STAT5A enhanced the phagocytosis and killing function of macrophages.** (A) WT and STAT5A-iMac *in vitro* killing assay against luciferase-expressing K562 and HO8910 cells with 10:1 a ratio for 48 h. *n* = 3. (B) Flow cytometry showing phagocytosis of K562 and HO8910 cells by WT and STAT5A-iMac cells. *n* = 3. (C) qRT–PCR showing differences in mRNA expression between WT and STAT5A-iMac. *n* = 3. (D) ELISA assay showing cytokine secretion in the medium between WT and STAT5A-iMac. *n* = 4. (E) Western blotting showing phosphorylation of ERK, NF-κB P65 and perforin in WT and STAT5A-iMac.

Next, we explored whether the NK-related transcription factors could promote other immune cell type to gain NK-related functions. We chose macrophage cells because they represent the separate lineage of myeloid cells and can be robustly differentiated from iPSCs. Based on our bioinformatics analysis, STAT5A plays an important role in immune cell lineage development, especially NK cells. Next, we explored whether STAT5A could promote macrophages to gain certain features and functions of NK cells. We transduced the STAT5A lentivirus to iPSCs (Fig. S6A and S6B), and we verified that STAT5A has no side effect on the pluripotency of iPSC (Fig. S6D). Upon differentiation to macrophage cells (iMacs), we examined their function with STAT5A overexpression. Firstly, we incubated the STAT5A-iMac cells or control iMac cells with blood cancer cell K562 and solid tumor cell HO8910. Compared with control cells, STAT5A-iMac showed increased killing activity and phagocytosis in both types of cancer cells (Fig. 6A and 6B). Based on this result, we examined the related proinflammatory factors, and found that STAT5A-iMac was significantly upregulated compared with WT-iMac gene expression of related to proinflammatory M1 cytokines such as *IL1A*, *IL1B*, and *TNF* (Fig. 6C), NK cells related cytokines and receptors such as *PRF1*, *IFNG*, *GZMB*, *NCR1*, and *NCR3* (Fig. 6C), and some metabolic genes in glycolysis associated with effector cell functions such as *PDK1*, *PDK2*, *HK2*, and *PFK2* (Fig. 6C). We also measured the NK cell cytolytic factor perforin as well as IL-12, IL-1α, and CCL-2 secreted in the medium, and found STAT5A-iMac cells had higher levels of these secreted factors (Fig. 6D). Accordingly, intracellular perforin was also upregulated (Fig. 6E). When we examined intracellular signaling pathways, we found phosphorylation levels of ERK and p-65 were also elevated (Fig. 6E). In conclusion, STAT5A boosted the cytotoxicity and phagocytosis capacity of macrophages, promoted the secretion of factors related to M1-like macrophages, and enhanced the release of NK cell-associated factors as well, notably perforin—a critical cytolytic agent in NK cell activity.

## Discussion

iPSC-derived immune cells have demonstrated immense potential for clinical application and present a sustainable, standardizable therapeutic production pathway [1, 2, 8]. Anticancer iPSC-derived NK cells have been explored in recent years; however, the production and manipulation of NK cells have been challenging due to their mysterious path of differentiation. In this study, we unveiled fetal NK development and iNK differentiation in a parallel and stagewise manner through a systems and network biology analysis of their dynamic transcriptomes.

Firstly, we found that distinct iPSC clones showed varying levels of iNK productivity, and productive clones had transcriptomic hallmarks of hematopoietic lineage priming. This finding indicates that iNK production could be improved even before the initiation of the differentiation protocol by optimizing the status of iPSCs so that an optimal level of hematopoietic priming is achieved and maintained.

To delineate the pivotal factors in differentiation, we investigated the progression of iNK development via scRNA-seq. A detailed developmental path from iPSC to a multilymphoid precursor and then to mature iNK cells and contaminated mast cells were delineated. After completion of differentiation, we identified two groups of iNK precursors by their unique cell cycle features and their lack of mature NK markers. This indicates the need for novel strategies that push these populations into a more mature state at the end of differentiation.

Using TF activity and network analysis, we revealed the role of classical regulators and discovered novel TFs controlling the complex trajectory of NK development. Collectively, we have drawn a high-resolution single-cell transcriptomic map that details further subclusters during iPSC to NK cell development as well as their transcriptional similarity to adult or fetal-derived NK cells. This analysis will provide a valuable blueprint to guide further bioengineering to more efficiently obtain iPSC-derived NK cells for immunotherapies.

As a critical downstream responder of the IL-2 and IL-15 signaling pathways, STAT5 is essential for the survival, development and maturation of NK cells. Specifically, NK cells deficient in STAT5A exhibited decreased levels of cyclin D2 and c-MYC, which are pivotal for cell cycle progression and growth [28]. Additionally, the lack of STAT5 also downregulated the expression of key effector molecules, such as perforin, granzyme A/B, and IFN-γ, which might account for the poor cytotoxic performance of STAT5-deficient NK cells against cancer cell lines [28]. A recent study further dissected the role of STAT5 in NK cell biology, unveiling that while STAT5 dimers were sufficient for early cell cycle progression of NK cells in response to IL-2 or IL-15, STAT5 tetramers were indispensable for normal expression of BCL2, an antiapoptotic factor. Loss of STAT5 tetramer in NK cells leads to an increased vulnerability to apoptosis following cytokine deprivation, highlighting the importance of STAT5 tetramers in maintaining NK cell viability and functionality [29]. Among the selected TFs, STAT5A was identified as having more specific activities in NK cells compared to others, suggesting it may play a significant role in NK cell differentiation. Therefore, we selected it for subsequent functional studies.

**Figure 7.**
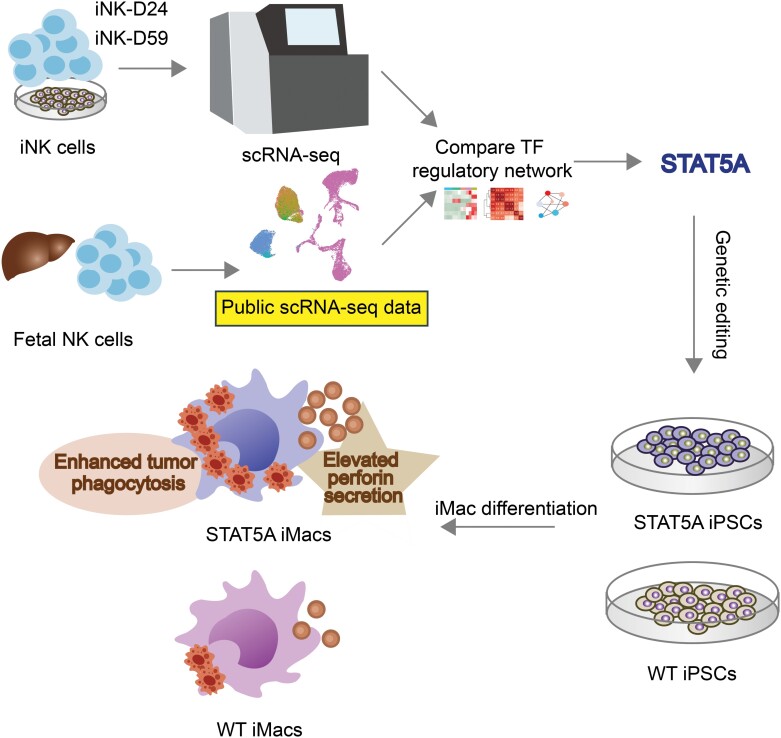
Graphical summary.

To investigate the role of STAT5A from a bioengineering and synthetic biology perspective, we applied it to macrophage differentiation from iPSCs. Our functional studies revealed that STAT5A boosts macrophage capabilities in both killing and phagocytosis and enhances the release of NK cell-associated factors, notably perforin—a critical cytolytic agent in NK cell activity (Fig. 7). This indicates that augmenting immune cells with key factors from different cell types can improve their overall functionality. Such an approach potentially broadens the scope of synthetic immune cells, offering new avenues and strategies in immunotherapy, thereby expanding the range of clinical applications.

## Research limitations

Although we revealed the role of classical regulators and discovered novel TFs controlling the complex trajectory of NK development, and identified the function of STAT5A in iMacs. The other roles of TFs have not been extensively studied, especially in NK cells. Further studies are needed to investigate the mechanisms of TFs in immune cells during development.

## Methods

### Cell culture

Human iPSCs were generated from a healthy donor and plated on Matrigel (354277, Corning) plates with mTeSR medium (85851, 85852, STEMCELL Technologies) as previously described [2]. ASPC-1 and K562 cells were cultured in RPMI1640 (SH30809, HyClone) medium, with 10% FBS (10099-141, Gibco), 100 µm nonessential amino acids (GNM71450, Gibco) and 100 U/mL penicillin, 100 µg/mL streptomycin (15140-122, Gibco).

### NK cell differentiation from iPSCs

iPSCs were dissociated with TrypLE (Gibco) to single cells when cells became 80%–90% confluent. It was then terminated with 1 mL mTeSR medium (#85850, STEMCELL Technologies), and cells were transferred to a new tube and centrifuged at 200 × *g* for 5 min. Cells were then resuspended using APEL media containing BMP4 (20 ng/mL, PeproTech), SCF (40 ng/mL, PeproTech), VEGF (20 ng/mL, PeproTech) and 10 μm Y-27632 (STEMCELL Technologies). Cells were counted and diluted to 80,000 cells/mL, and seeded at 8000 cells/well in the 96-well low attached plates. The plates were centrifuged at 300 × *g* for 5 min to form EBs, and cultured for the following 6 days. On Day 7, 20–30 EBs were plated onto one well of six-well plate precoated with Matrigel (354277, Corning), and the medium was switched to StemSpan^™^-XF medium (100-0037, STEMCELL Technologies) with IL-3 (5 ng/mL, PeproTech), SCF (20 ng/mL, PeproTech), IL-7 (20 ng/mL, PeproTech), IL-15 (10 ng/mL, PeproTech) and Flt3 ligand (10 ng/mL, PeproTech). After the first 2 week of the differentiation, IL-3 was removed from the indicated differentiation culture media. NK cells were harvested at approximately from 20 days.

### Flow cytometry analysis

Cells were harvested and digested into single-cell suspension, and then washed with DPBS and stained with antibodies for 15 min at room temperature or 4°C. Flow cytometry antibodies: CD34 (301330, BioLegend), CD45 (333606, BioLegend), CD56 (374204, BioLegend), NKG2D (361715, BioLegend), NKp46 (331917, BioLegend), NKp30 (325213, BioLegend) All flow cytometry experiments were done on BD Fortessa and the data was analyzed with the FlowJo software.

### Cytotoxicity assays

Luciferase-expressing cancer cells were cultured in RPMI culture medium supplemented with 10% FBS. The effector NK and macrophage cells were added with various effector:target (E:T) ratios as indicated, and then chemiluminescence was measured by a microplate reader. NK and macrophage cell cytotoxicity (%) was calculated as the following: NK cell cytotoxicity (%) = [1 − (measured value − control value)/(maximum value − control value)] × 100. The control value represents the fluorescence value of the well without cells, and the maximum value represents the fluorescence value of the well with only cancer cells.

### Phagocytosis assay

Tumor cells of tdTomato-K562 or HO8910 were co-cultured with iPSC differentiated macrophage cells lively in a 1:10 ratio. After 24 h, the cells were washed with PBS, treated and lifted by TrypLE Selected Enzyme, then stained with CD11b-APC antibodies for 15 min at room temperature and analyzed by flow cytometry. Double-positive areas represent tumor cells that are engulfed by macrophages.

### Bulk RNA-seq

Briefly, total RNA was extracted using FastPure Cell/Tissue Total RNA Isolation Kit V2 (Vazyme, RC112-01) from cells for iPSC, EB, iNK at day 26, iNK at day 59 and NK form PBMC. The library was constructed with Universal V8 RNA-seq Library Prep Kit for Illumina (Vazyme, NR605-01). The constructed library at the size of 400–600 bp was sequenced on an Illumina NextSeq 500.

### scRNA-seq

Differentiated iNK cells at Days 24 and 59 were collected, respectively. Then cells were resuspended with precold PBS containing 0.05% BSA and passed through a 40 μm filter to obtain single cells. The single-cell RNA-seq experiment was performed on the 10× Genomics platform according to instructions [30]. Due to insufficient samples of early iNK cells from the same batch, we used Days 24 and 26 for scRNA-seq and bulk RNA-seq, respectively.

### Processing and analysis of scRNA-seq data

Single-cell data from iNK samples were aligned using the Cell Ranger pipeline. The resultant counts were further processed in accordance with the Seurat v4 pipeline215 [31]. The cell type of each cluster in these samples was found using the SciBet algorithm and the fetal liver dataset from Popescu et al. as a reference set [9, 32]. Cell types were then assigned based on the SciBet predictions or in the case of equivocal results, a combination of SciBet and Seurat identified marker genes’ expression. Clusters that contained the sample cell type were merged to create one cluster per cell type. Seurat positively expressed marker genes were also used to identify enriched pathways in each cell type using gene set enrichment analysis.

In accordance with the benchmark review by Saelens et al., we used the ScanPy and PAGA pipelines to derive pseudotime graphs for the iNK samples [33]. Connectivity maps were also constructed and overlaid onto the pseudotime maps to display the relationships between clusters.

### TF activity assessment

We assessed the activity of transcription factors in single cells by adapting the template-matching approach created by Pavlidis and Noble and the TF-target database, TRRUST v2 [34, 35]. This was done separately for each iNK dataset and for select cell types within the fetal liver dataset. We started by normalizing expression for every gene in the TRRUST database so that each gene fits a normal distribution between −1 and 1. A template is then defined for each TF in TRRUST with genes predicted to be activated defined as 1 and genes predicted to be repressed being defined as −1. Each cell’s adherence to each template was then measured by Pearson correlation. This results in a TF activity measurement for each TF in each cell which varies from –1 to 1. Thus –1 represents the ideal cell with the TF maximally repressed, and conversely 1 represents the ideally activated cell by the TF in question. Because normalization was completed for each dataset separately, only within-dataset comparisons are appropriate.

### Comparison of *in vivo* verses *in vitro* NK cells

Adult NK cell scRNA-seq from Crinier et al. and fetal liver hematopoietic cells were merged with the iNK-24 and iNK-59 datasets, and batch effects were removed using Harmony within Seurat [36]. Non-NK cells were recategorized as “Non-NK” to aid in visualization and new UMAP visualizations were generated. The correlation of the average expression profiles for all cell types was measured and plotted to show subpopulation associations. NK-relevant pathways were selected from the GO database and the *Z*-score for genes within those pathways were plotted to show NK cell phenotypes across the merged datasets. Gene set enrichment analysis was run on the Seurat marker genes extracted from the iNK-59 dataset to characterize their biological phenotypes.

### Visualization

Network visualizations were rendered using the igraph R package and Cytoscape. Heatmap representations were rendered using ComplexHeatmap in R. Non-heatmap or Seurat figures were rendered using ggplot2 in R.

### Statistical analysis

All statistical tests were performed using the GraphPad Prism software (V.8.3.0). Data is represented as the mean ± SD. Statistics by two-tailed Student’s *t*-test, statistical significance is shown as *P < 0.05, **P < 0.01, ***P < 0.001, and ****P < 0.0001.

### Research ethics

The iNK, WT-iMac, and STAT5A- iMac are derived from human pluripotent stem cells via direct *in vitro* differentiation and gene editing established by previous study. There are no relevant ethical issues.

## Supplementary Material

lnae032_suppl_Supplementary_Materials

## Data Availability

All data needed to evaluate the conclusions in the paper are present in the paper and/or the Supplementary Materials. Additional data related to this paper can be requested from the authors.
