# Supplementary material for: Comparative dissection of transcriptional landscapes of human iPSC-NK differentiation and NK cell development
Source: Life Med. 2024 Sep 6;3(4):lnae032. doi: 10.1093/lifemedi/lnae032 (PMC11749552; doi:10.1093/lifemedi/lnae032)

Supplementary data

Figure S1

1. Flow cytometry shows variation of CD34 expression among different iPSC clones.
2. Volcano plot compares gene expression of iPSC 1-1 and iPSC 3-8
3. Top GO terms enriched in genes up-regulated on iPSC 1-1 compared with iPSC 3-8

(D-J) qRT-PCR shows pluripotency markers, HSC markers, NK cytotoxicity markers, KIRs, cytokine receptors, immune receptors and check point markers expression in iPSCs, EB, iNK-D26, iNK-D59 and PBMC-NK.

(K) Expression of NK-related activating receptors from RNA-seq.

Figure S2

(A) Volcano plot compares gene expression of iNK-D59 and iPSC 3-8.

(B) Volcano plot compares gene expression of iNK-D26 and EB.

(C) Volcano plot compares gene expression of iNK-D59 and iNK-D26.

(D) Heatmap shows highly dynamic genes expression during iNK differentiation.

Figure S3

(A) Expression of featured markers expression in certain clusters at iNK day 24 on UMAP.

(B) Expression of NK markers and *KIT* expression at iNK day 59 on UMAP.

(C) The major GO Biological Process terms for c0, c3 and c4 clusters in iNK day 59.

(D) Partition-based graph abstraction (PAGA) topology tree of cell clusters at iNK day 24. The width of line in PAGA indicates the strength of connectivity between cell clusters.

(E) Partition-based graph abstraction (PAGA) topology tree of cell clusters at iNK day 59. The width of line in PAGA indicates the strength of connectivity between cell clusters.

(F) Violin plots show the expression level of *KLRD1* and *KIT* in NK cells and other subgroups

(G) Dot plots showing the expression levels of *KLRD1* and *KIT* in defined NK clusters.

Figure S4

(A) Heatmap shows differential expressed genes of adult, fetal and iPSC derived NK cells.

(B) The GO Biological Process terms for NK cells from different sources.

Figure S5

(A) Box plots show the activity score of *SPI1*, *GATA3* and *ZBTB16* at developmental timing with published *in vivo* scRNA-seq data.

(B) Box plots show the activity score of lineage specific TFs with published *in vivo* scRNA-seq data.

(C) Heatmap shows the negative regulation TF activity at day 24 differentiated NK cells and other-related clusters. Colors represent the average TF activity.

(D) Heatmap shows the negative regulation TF activity at day 59 differentiated NK cells compared with mast cells. Colors represent the average TF activity.

Figure S6

(A) Microscopic pictures showing the overexpression of STAT5A in iPSCs.

(B) Western blotting showing the overexpression of STAT5A in iPSCs.

(C) Confocal microscopic images showing the overexpression of STAT5A in iPSCs.

(D) Flow cytometry showing the pluripotency of iPSC that overexpresses STAT5A.

(E) qRT-PCR and western blotting showing showing the overexpression of STAT5A in iMac.


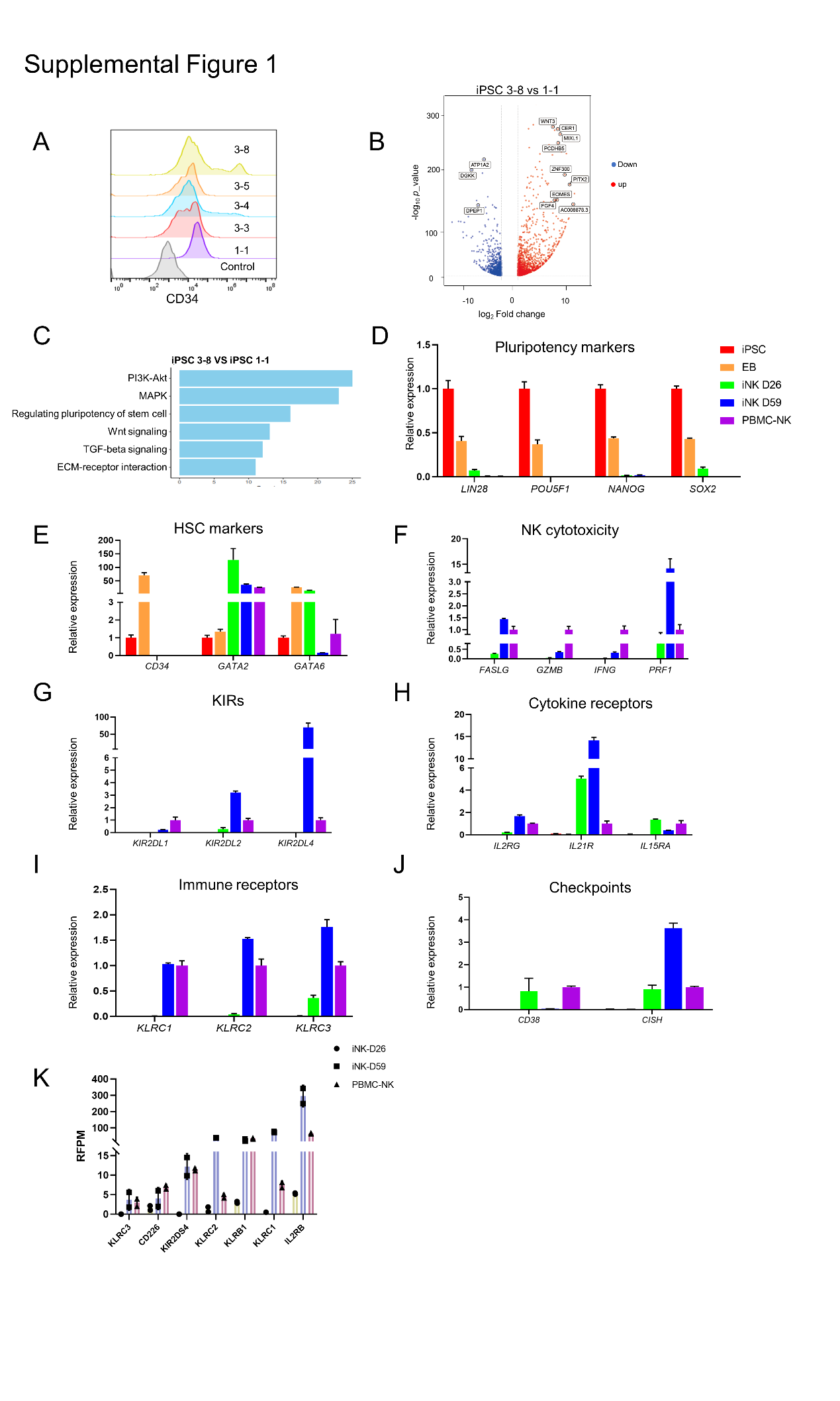


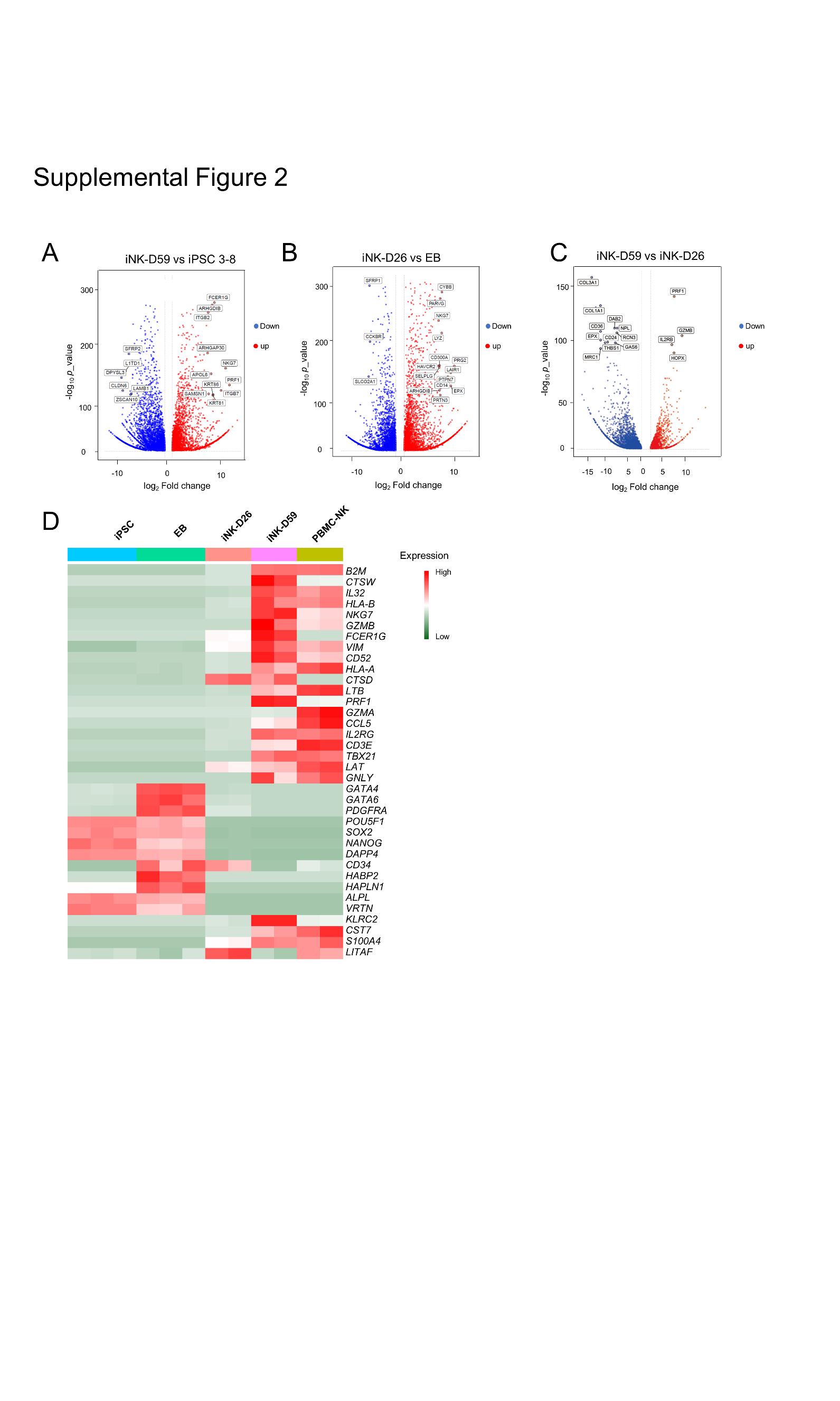


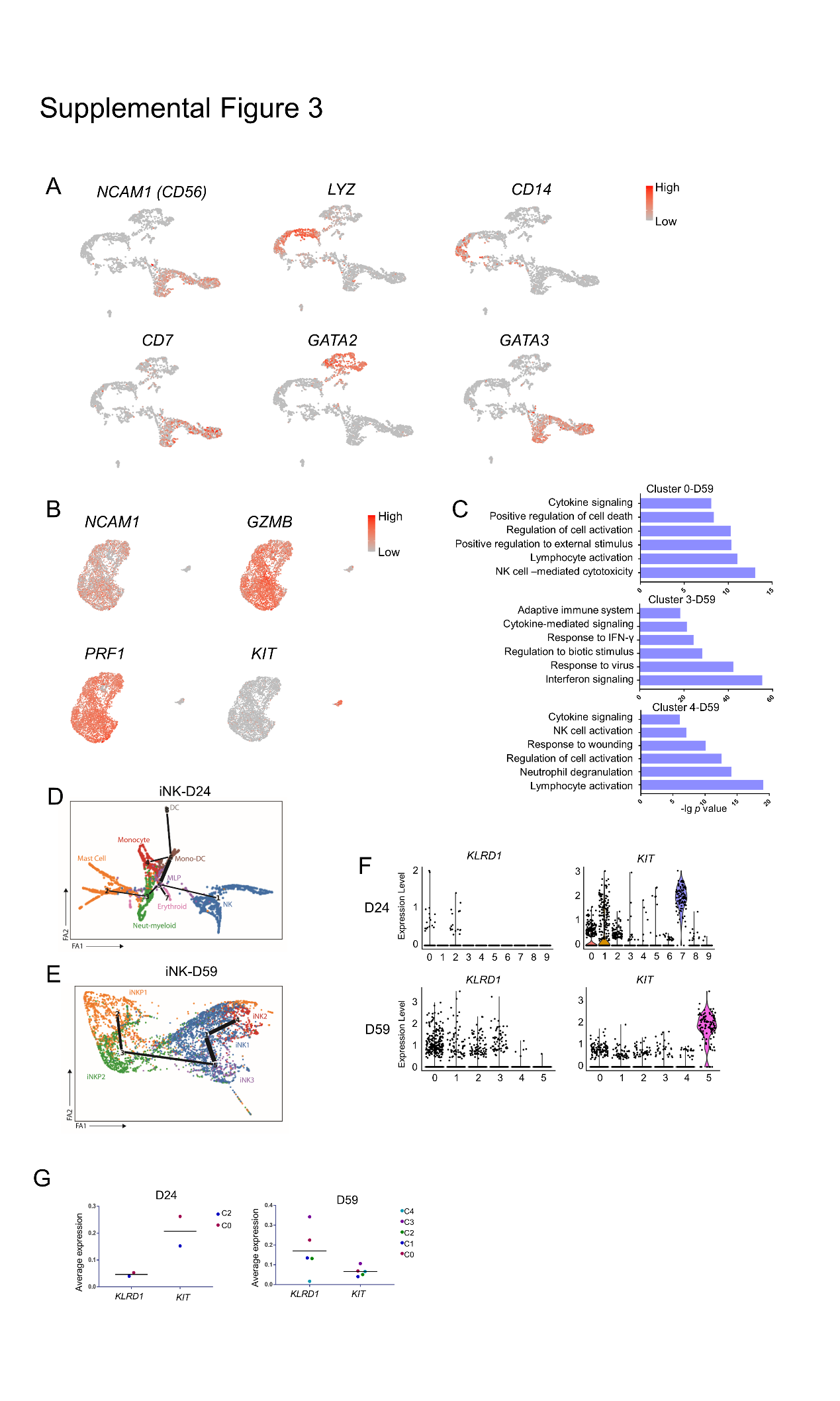


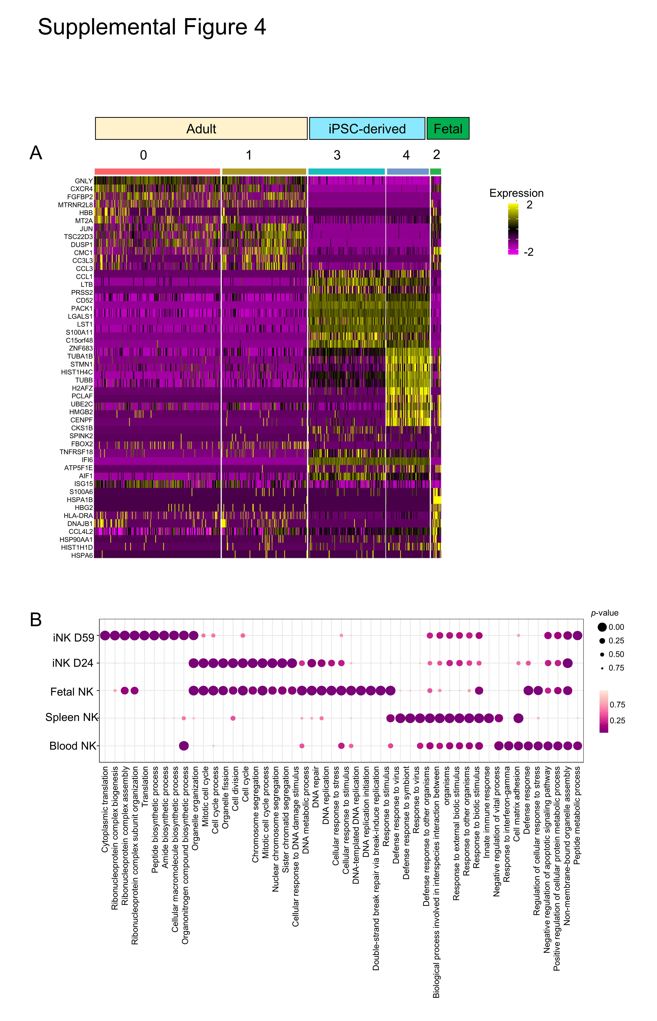


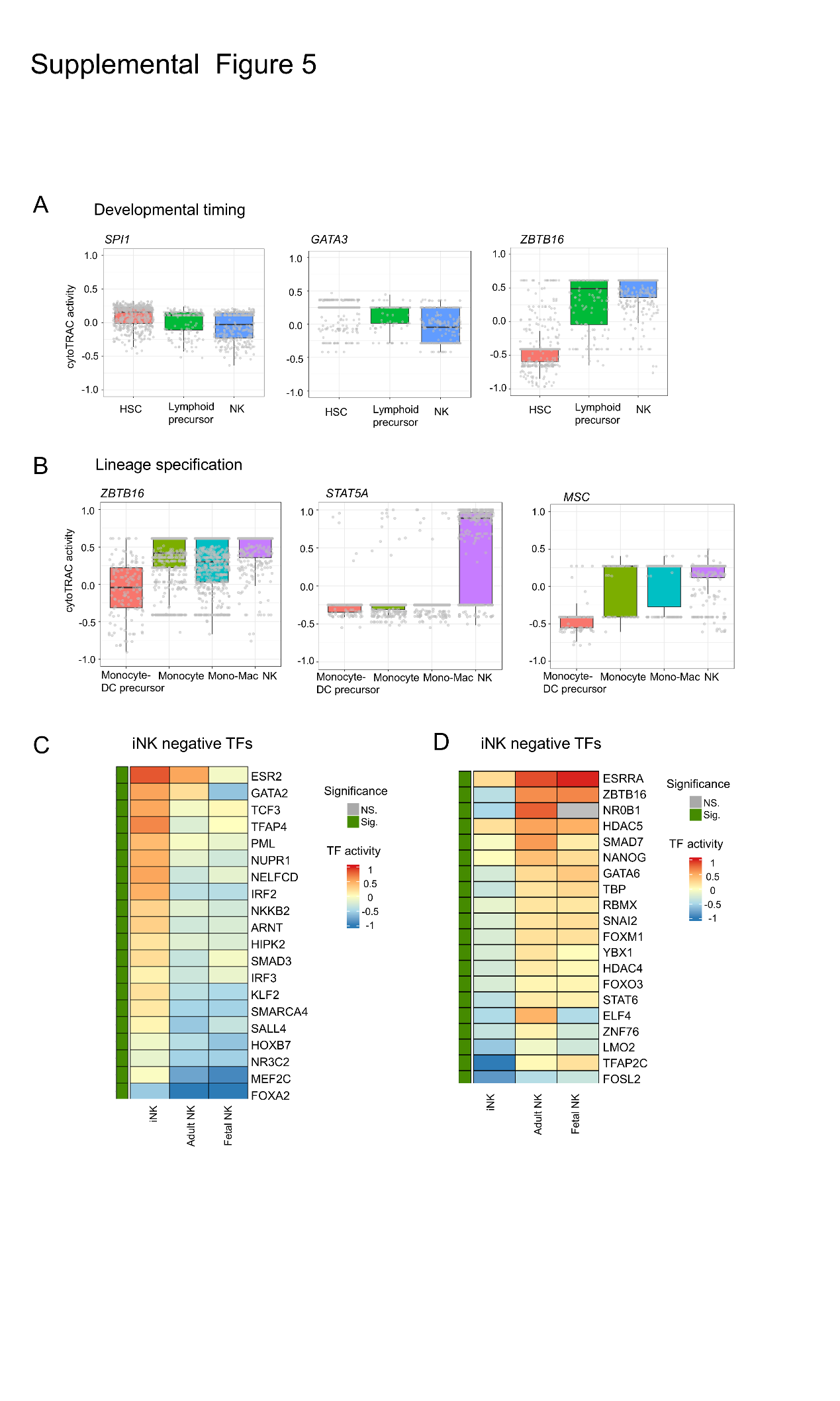


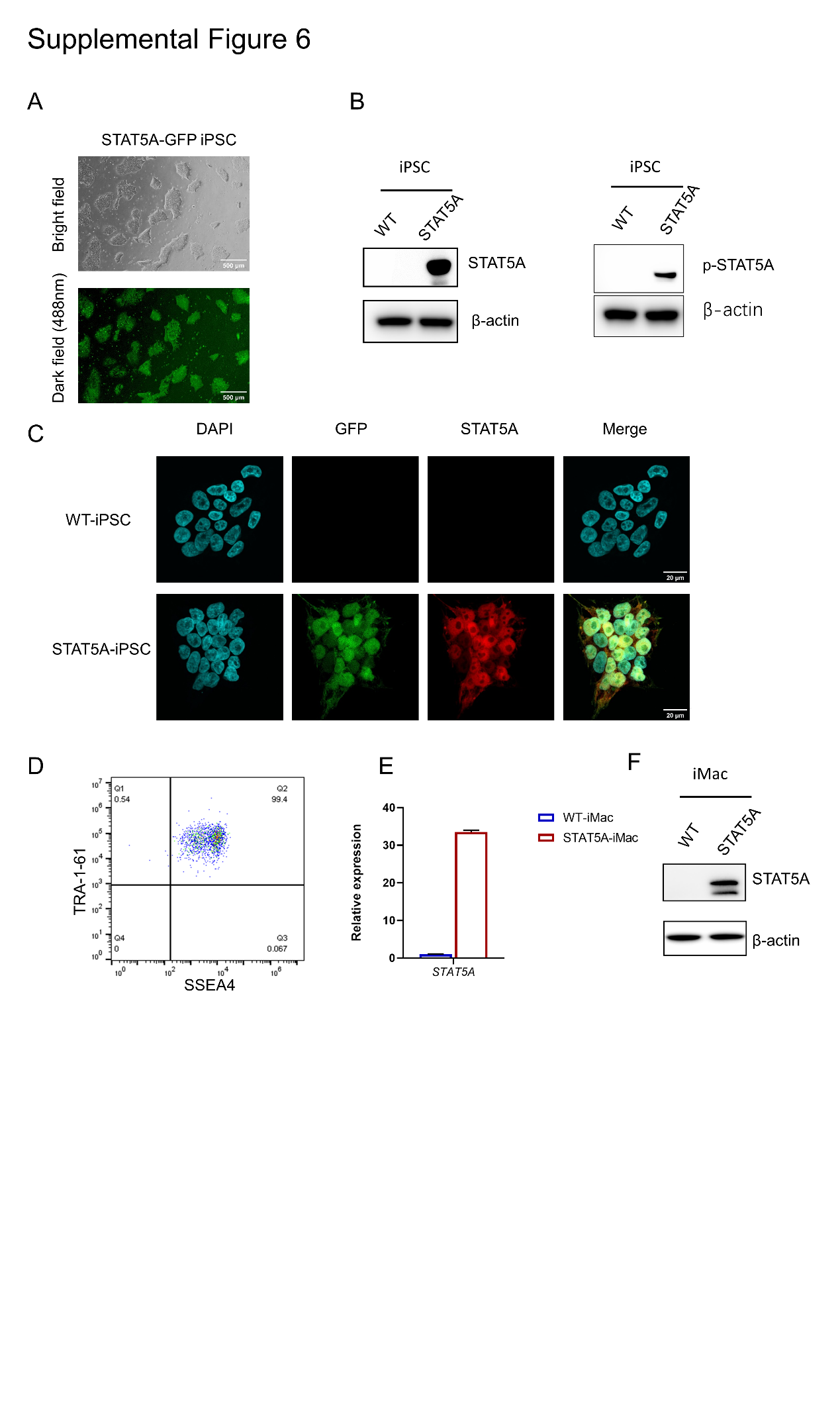

Supplement: lnae032_suppl_Supplementary_Materials [file lnae032_suppl_supplementary_materials.docx]
